# The Universal Eating Monitor (UEM): objective assessment of food intake behavior in the laboratory setting

**DOI:** 10.1038/s41366-022-01089-0

**Published:** 2022-03-01

**Authors:** Harry R. Kissileff

**Affiliations:** Mount Sinai Morningside Hospital and Department of Medicine Icahn School of Medicine, 1111 Amsterdam Ave., New York, NY 10025 USA

**Keywords:** Translational research, Preclinical research

## Abstract

The Universal Eating Monitor was a term used to describe a device used in a laboratory setting that enabled investigators to measure, with the same instrument, the rate of eating either solids or liquids, hence the term “universal”. It consisted of an electronic balance placed in a false panel under a table cloth on which could be placed a food reservoir that contained either solid or liquefied food. The device was created in order to determine whether rates of eating differed in pattern between solid and liquid foods. An acceptable mixture of foods of identical composition that could be served as either solid or blended as a liquid was used to test the hypothesis that eating rate and intake were affected by physical composition. A best-fitting mathematical function (intake was quadratic function of time, with coefficients varying among foods used and experimental conditions), quantified intake rates. The device was used to test a variety of mechanisms underlying food intake control. Eating rates were linear when solid foods were used, but negatively accelerated with liquids. Overall, intake did not differ between solid and liquefied food of identical composition. Satiation on a calorie for calorie basis was different among foods, but physical composition interacted with energy density. Hormones and gastric distension were strong influences on food intake and rate of eating. Individuals with bulimia nervosa and binge eating disorder ate more than individuals without these disturbances. Intake in social and individual contexts was identical, but the rate of eating was slower when two individuals dined together. The eating monitor has been a useful instrument for elucidating controls of food intake and describing eating pathology.

## Introduction

*Overview:* The original ultimate goal of the studies reviewed in this paper was to use the laboratory test meal, measured with the UEM [1], to translate animal models of ingestion to humans for the study of the physiological controls of food intake under standardized conditions. As noted by Booth [2] “Measures of intake alone will not provide evidence for the control of intake”, because the mechanisms being translated are ingestive acts that result in intake. Amounts (usually in gravimetric and volumetric) units consumed per unit time (rates) are the result of all sources of influences that operate moment to moment during a bout of ingestion. The UEM was initially validated as a measure of eating rate by comparison of experimental influences on the “momentary rates of consumption compared between foods and…contexts” (see p. 65) [3], These validations are best expressed as dose-effect responses of rates of eating regressed mathematically from graded variations in experimentally manipulated variables. However, as I progressed through this review and read widely, I realized that the microstructure of eating in humans is only partially controlled by physiology. Hence this review includes influences on eating rates from context, culture, social relations, personality, body mass index, surgery, environment, and physical composition of the items being ingested.

In the original description [1] of the UEM and in two subsequent reviews [4, 5] I described animal and human precursors of the UEM. A historical background on measures of rates of eating from cumulative intake curves was developed a few years after the initial description [6]. Articles for this paper were selected for review from: (1) a google search of all articles that cited the UEM, (2) all articles that appeared in a search for UEM. I do not claim that the search was exhaustive, but it is sufficient to address the critical variables I believe are in need of review. Consequently. this review identifies critical variables addressed by the UEM, its strengths and limitations as an experimental tool for study of obesity, and the need for adaptation of the methodology for analyzing the data [7]. Several other reviews of eating monitor technology and its application have also been published [8–11].

*Description of the UEM and the initial study*: The UEM [1] is a device used by investigators of appetite and food intake in humans to measure rates of eating, and scaled responses to appetite-related feelings (fullness, satisfaction, etc), of both solid, liquefied foods and beverages. It consists of an electronic balance placed on a table with a false panel in the top covered by a tablecloth and concealed to participants. As food is consumed from a bowl placed on top of the panel, its decreasing weight is transmitted to a computer in an adjacent room, and the disappearing weight is converted by the computer to a curve of intake vs. time, which can be fitted to an equation for analysis.

The UEM was developed to compare the satiating influences and rates of eating of solid and liquefied food, because, in 1980, it was unclear which physical consistency in foods would be more effective for weight control, and whether rate of eating was a determinant of amount consumed. Solids and liquids have different physiological effects related both to their rates of consumption, oral and post-oral processing. In an earlier study [12], equicaloric portions of liquids and solids given to human subjects induced the same intake from both consistencies, and the interpretation was that physical consistency did not matter. However, the outcome was confounded by differences in nutrient composition, as well as consistency. The UEM was designed along with a novel food mixture, as opposed to a formula, that could be served in either solid or liquefied form. The food consisted of a yogurt, fruit, and nut combination, whose components were simply mixed (solid, chewable version) or blended in a food processor (liquefied version). The influence of visual cues could be assessed by hiding the reservoir. The device was then applied to investigations of physiological mechanisms that control eating in healthy participants and individuals with a variety of medical problems.

## Critical variables needed to create a physiological eating test

I identified at least six critical variables (see below) which prior studies had not fully considered in their attempts to compare types of individuals and physiological manipulations.

- *Choice of food/beverage and standardized testing:* In our initial study [1] we wanted a food that could be served as a solid or liquid. Most of the previous work had been done on liquids, which were easier than solids to satisfy balanced nutritional criteria, and commercially available. Little attention was paid to how often such foods were consumed, how much participants “liked” them, and how these variables would impact the outcome (i.e., amount eaten or rate of consumption). For optimal results, groups of individuals, for planned studies of manipulations should be chosen on the following bases: (1) How much they like the foods or beverages as indicated by ratings on, or efforts to consume, them (see [13] for differences in ratings between and within subjects); (2) How frequently they consume the item, and how to select groups of participants and items as uniformly as possible; (3) What properties of the item are appropriate to the manipulation (i.e., taste, physical consistency, nutrient content, energy density, etc). For some clinical studies in which a single trial needs to suffice for diagnostic or evaluative purposes, frequently there is no standardized food. Offering each person his or her favorite, preferred, or usually eaten food is not a solution, since variations in individual choice will be completely confounded with any eating behavior characteristic of the individual the test is supposed to reveal. The best one can do under these conditions is to utilize foods that are most likely consumed by the group under study. Then one can evaluate the potential impact of individual preference/frequency variations on the measured outcomes. Finally, the participant should be tested in a standardized metabolic state to the extent possible. In our studies, we used a 300 kcal food combination given 2 to 3 h before the main test [1]. It is not a good idea to tailor any treatment variable to individual subjects, since such a procedure confounds treatment and subject sources of variance. If a subject variable is suspected to influence treatment outcome, it should be added as a covariate, and hence becomes a moderator.
- *Multi- vs single item meals*. A more serious problem with interpretation of test meals occurs when instead of a single item, multiple items are presented at the same time. While it is natural in both animals and humans for ingestive bouts to contain multiple items, the problem of how to combine the items, and the confounding influences of the order of consumption, makes it difficult to obtain satisfactory answers to questions about mechanisms of meal size or rate of eating control under these conditions. Typically, total weight, energy contents, or macronutrient amounts are presented with good reasons for each, but the outcome is the same: It is difficult determine underlying mechanisms in multiple item meals without potential confounders. One attempt [14] to measure multiple components that determine meal size was done by the experimenter giving participants four courses in succession. Three courses (1,3, and 4) were limited in size and therefore effectively fixed. The second course was abundant with ad libitum intake allowed. The rates of eating the courses were independent of one another and did not reflect a cumulative effect. The rates of eating indicated by curve parameters did not predict intake. Hence the authors concluded that the parameters reflected long-term cognitive effects rather than regulatory processes. The potential indication of curve coefficients for food intake physiological processes requires that these processes be directly manipulated. However, because consumatory behavior is always under simultaneous long and short term influence it is difficult to measure these controls independently.
- *Reliable measuring device:* The UEM is a reliable measuring instrument, because consistent results are obtained with repeated trials under the same conditions [6]. The day-to-day variation within individuals averaged ± 15%. Obviously, reliability is important, and any new devices should be tested with at least eight, and probably more, individuals for at least four non-consecutive day trials.
- *Mathematical expression of rate:* Mathematical models of cumulative intake curves were developed in order to test underlying assumptions about the causal determinants of eating rate. If parameters of individual curves represent common underlying events, then variability in these parameters should reflect underlying controls which can be statistically modeled and tested. Before, we selected the quadratic as an important theoretical and practical solution to mathematical expression of rate of eating, we reviewed previously used models for cumulative intakes, and described their theoretical properties. The quadratic model was proposed to reflect two sorts of processes, an excitatory, and an inhibitory [15]. It was easier to obtain coefficients for the quadratic than exponential model [16]. The coefficients also had a potential physiological underpinning which was simpler than, but consistent with the exponential model of Davis and Levine and the theory of Stellar [15, 17]. The Davis and Levine model employed a series of constants that could be reduced to two, one of which was related to the initial rate of eating, while the other was related to the slowing of the rate.
- *Interpretation of the coefficients*: The coefficients were interpreted as facilitating and inhibiting [18]. However, the rate of deceleration (the inhibitory component) and initial rate (the facilitatory) were significantly correlated and hence were not independent. Nevertheless, our proposal that initial rates were facilitatory and deceleration rates inhibitory, has been confirmed partially, by subsequent work on the licking behavior in animals. “Initial rate of ingestion (the intercept) *measures* palatability and that the slope constant is a measure of the rate of development of a negative feedback satiety signal” [19].
  We now note that the coefficients of the differentiated quadratic equation (rate = a – bt) exhibited identical properties (i.e., additivity in log units) to natural log-transformed exponential rate (dy/dt = Ae^−bt^) is ln(Ae^−bt^) = ln(A) – bt, which is log-transformed intercept and an apparently linear slope, but which in actuality is a fraction and not a difference. Note dy/dt is the derivative of intake with respect to time, A and a are intercepts, i.e., initial value, of the quadratic equation for rate of intake vs time, e is the base of the natural logarithms, b is the slope of the relation between intake and time, t is time and ln is natural logarithm The difference between these models is that rate of eating derived from the quadratic cumulative intake is a constant difference across time, whereas the rate of eating from the exponential model is constant ratio of each rate from the current rate across time (i.e., a difference of logs). A constant reduction implies that the inhibitory signal is a constant, whereas the exponential model implies that inhibition is gradually increasing in proportion to its current level. Both models require a termination signal, independent of the rates. In actual tests of the models, we ended up fitting the quadratic to cumulative intake curves because it was easier, and we found that intake stopped well before the balance of excitation and inhibition (i.e., the maximum of the quadratic or the derivative reach zero) was obtained. As a result, we added a threshold variable to our model (see “Gate control” in figure 2 from [18]).
  Recently [7] a three component model has been proposed that employed a differential equation in which there is an initial acceleration followed by deceleration. We have rarely seen this early acceleration followed by deceleration in our data, but where it is present, the model could be used. However, a linearizable equivalent, the cubic, has also been proposed [20] to deal with curves that have more than one inflection point, and while Thomas et al. [7] compared their model to a quadratic, it should have been compared to a cubic, because a cubic has three parameters.
- *Instructions to participants*: The amounts consumed and rates of eating are critically dependent on instructions to participants and need to be tailored to the objectives of the study. Intakes of the same items depend on the context of the instructions and can vary from minute portions eaten solely for rating [21] through eating as much as you like [1] or to feel comfortably satisfied to eating to capacity [22, 23]. The reason instructions affect intake is that they are able to estimate and report accurately the size of a portion they can eat in a given context [24, 25].
  Recently the “capacity meal” (i.e., a meal in which the participant was instructed to eat to capacity) has also been used as a predictor of success after obesity surgery [26]. The hypothesis was that larger capacity meals signal less sensitivity to satiation signals and thereby poorer outcome. This interesting hypothesis deserves further testing, but the size of a meal alone is not sufficient to indicate a satiation disturbance as noted in our studies on patients with binge eating disorder [23].
- *Reactions, expectations, cover stories, and manipulation checks*: An important critical factor in studies of human food intake is the potential that the participant’s knowledge of the study could influence the outcome, particularly in patients with obesity [27]. Furthermore social factors have been shown to be an important influence on food consumption and persons with obesity reacted differently than controls to eating situations designed to test motivational factors [28–30]. In order to avoid possible influence of the knowledge that their food intake was being monitored on a second to second basis, the participants were told that we were interested in their reactions to the food after they had eaten it. To fulfill this “cover story” we gave them rating sheets to fill out after they had stopped eating. We also used those ratings to determine whether stopping was caused by satisfaction or discomfort.
- *Reproducibility of conditions*: The use of the UEM in a uniform environment with the same instructions under a variety of experimental manipulations, provided a framework for testing potential mechanisms for reduction of eating that could be applied to treatment of obesity and/or eating disorders. These included drugs, hormones, food items, as well as controls of mindset or context, and in particular instructions to eat a certain amount or at a certain rate. The use of UEM in a controlled testing environment enabled precise measurement of the influences of these variables.

## Initial findings (1980–2001) and subsequent developments in relation to theory

The findings with the UEM fall into three sections: (a) Predictions from the cumulative intake coefficients and rates of eating, (b) Effects on intake only, (c) Study of satiation scaling and eating disorders.

*Predictions from the cumulative intake coefficients and rates of eating*: The initial findings related to cumulative intakes and microstructure have been reviewed [4, 5]. The hypothesis that the cumulative intake curve could be used to discriminate eating problems in obesity and partition controls into two types, facilitation and inhibition turned out to be a mixed bag. With solid and liquefied versions of a food of identical composition, the initial rate of eating the liquefied version was faster, but also slowed more quickly than the solid [1]. With a large 2 quart container as the reservoir, we found no difference in intake or cumulative intake curve coefficients whether the container was covered or not. Initial rates of eating were marginally faster for men (118 g/min) than for women (74 g/min, F_1,12_ = 4.25, *p* = .06). We concluded that visual cues were not important in this situation. Meyer and Pudel’s [27, 31–33] hypothesis that individuals with obesity failed to exhibit normal (i.e., negatively accelerated) cumulative intake curves, could not be tested with our initial test food (yogurt and fruit), because the patients with obesity did not like it. With a more solid, but still relatively energy dilute, macaroni and beef, we found a variety of curves, and some curves were not at all smooth [5]. The Meyer-Pudel hypothesis of uniform failure to exhibit negatively accelerated curves was not confirmed, possibly owing to the difference in palatability between our liquid test food and theirs. In 20-20 hindsight, it would have been better to have developed a diet that was equally liked by both patients and controls. Nevertheless, later results have proven that classification by initial and decelerated eating rates is clinically useful (see Clinical applications below).

Food deprivation affected both coefficients (i.e., linear and quadratic [18]), while administration of cholecystokinin (CCK-8), shown to induce satiation in animals [34], affected neither coefficient, but simply terminated the meal sooner [35, 36]. A similar finding of no effect of satiation-inducing peptide on initial rate and rate of deceleration was found for GLP-1 [37]. In this case, meal duration was not significantly reduced, but overall eating rate was slower after peptide than saline. Like deprivation, adulteration of a sweet liquid diet with cumin which made it unpalatable for our cohort, reduced both the initial rate (linear coefficient) and the rate of deceleration as well [13]. However, patients with bulimia nervosa [22] consistently accelerated eating rates throughout the meal. The average rate of eating the ice-cream meal was higher in patients with bulimia than it those with obesity with or without binge eating disorder and healthy controls [22]. There were no differences in eating rates in the latter three groups [22]. The “preloading paradigm” [38] was used to compare the satiating influences of solids and liquids of different volumes and energy densities [39]. In these studies, an eating episode was divided into two portions, one fixed preload as a “dose” and the other consumed to satisfaction as the “response”. If the slope of a multi-dose preload was linear it was defined as the “satiation efficiency” [38]. The slope and intercept are useful metrics for comparison of the satiating effects of different foods, (or any graded manipulation), because they take into account both the constant (intercept) and variable (slope) effects of the manipulation of preloading itself, which other methods that utilize a compensation index do not [40].

The decrease in the initial rates, not the rates of deceleration, were the main determinants of intake reduction after the more satiating (i.e., intake inhibiting) treatment, thereby raising the question of whether changes in rates are really reflections of satiation, an interpretation that accords with the previous conclusions of Westerterp et al cited above [14]. This pattern of initial rate being more significantly affected than rate of deceleration persisted when the infusion of CCK-8 (2.25 µg) was combined with a large, (500 g) but not a small (100 g), preload of soup [41]. The large preload was essential for the effectiveness of CCK-8 in reducing intake of a semi-solid macaroni and beef meal by 227 g ± 72 SE (*p* = 0.002, *N* = 12), while reduction after the small preload (69 g ± 72 SE) was insignificant. The initial rate of eating this meal was also significantly reduced after the large preload and CCK (127 g/min) by 41 g/min ± 9.5 SE (*p* = 0.002) compared to the small (85 g/min), but the rate of deceleration was reduced, not increased, although the change did not reach significance. In any case, the rates could be useful indices of the influences of manipulations, even if their interpretations require more study.

*Effects on intake only or average rate with the UEM*: Studies were done to evaluate the effects of drugs, hormones, exercise, presence of companions, and food components, by means of within subject counterbalanced repeated measures designs. Bombesin and Glucagon [42], but not when combined with CCK, suppressed intake significantly compared to saline control. Insulin infusion, while glucose was held constant, did not reduce intake [43]. Phenylpropanolamine did not reduce intake, but increased mood [44]. Large doses (greater than 169 kcal), but not small (30 and 104 kcal), of psyllium [45] in a nutrient base did suppress intake (499 kcal, at low vs 411 kcal, at high). The effect appeared to be a threshold and not dose-effect, as the next higher dose (234 kcal) did not reduce intake more than the 169 kcal dose. There were significant effects of strenuous vs. moderate exercise, on food intake, between women with and without obesity [46]. Intake of a liquefied test meal (1.04 kcal/g) eaten I5 mm after exercise was significantly less after the strenuous (620 g) than after the moderate (754 g) exercise in the women without obesity, but was no different after the two conditions (532 g after strenuous, 58 1 g after moderate) in women with obesity. The presence of a companion eating the same food (a macaroni and beef meal) in a cafeteria setting did not change intake compared to pair eating separately in the laboratory, but the rate of eating was slower in the cafeteria [47]. The influence of a 1% vs 15% glucose preload was the same in both settings (141 g less after 15%). This result indicates that rate of eating can be uncoupled from amount consumed, and consequently rate is not a necessary determinant of meal size as envisioned by the excitatory and inhibitory model [18].

*Satiation scaling and eating disorders*: Because we observed an increased rate of eating and excessive meal sizes in patients with bulimia [22], we proposed that these phenomena were indicators of incomplete satiation. In order to obtain a more direct measure of how satiated the patient felt, we adapted the UEM to interrupt ongoing meals at discrete intake intervals at which they rated feelings that would be associated with satiation (i.e., how hungry, full, thirsty, felt they’d had enough) or with discomfort, pleasantness of the food, (mouth feelings) [48]. These feelings were then plotted against intake and rates of satiation per unit food were computed. Indeed, [49] more food was required to generate the same feeling of satiation in the patients with bulimia than in controls. Other laboratories adapted this procedure to investigate satiation and pleasantness of foods that differed in palatability [9]. Two additional studies were conducted with this methodology in patients with obesity and binge eating disorder [23, 50]. Since it could be argued that intake alone is the only variable that needs to be measured, it is important to note that these ratings are also essential to demonstrate subject compliance with the instructions, and that when subjects are instructed to eat until satisfied that they are not stopping from discomfort.

## Applications from Ingestive Behavior Core Laboratory at St. Luke’s/Roosevelt Hospital (since 2000–2001 reviews [4, 5])

*Rate of eating (2009)*: In order to test whether patients with bulimia consumed large meals because they ate faster, thereby by-passing satiation signals which require time to generate, a transparent cup with a line marked across the center was placed on the UEM. The test meal was pumped into the cup at a fixed rate controlled by the observer located in an adjacent room. The subject was told to eat at a rate that kept the level in the cup at the line. For the control group the mean difference in consumption between fast and slow rates was 168.9 g ± 53.2 SE (*p* < 0.05), whereas for patients it was only 10.8 g ± 54.8 SE (ns) when the rate of eating was increased by 70 g/min to 140 g/min. These results should be interpreted with caution, because there was a strong order effect with the large difference occurring when the slow rate was first. The issue of the role of rate of eating in the control of amount consumed has been reviewed in two important and excellent papers [51, 52].

*Leptin*: The UEM was used in a replicated 4-meal design to measure the effects of weight loss induced by leptin injection under two different eating conditions with two trials each before and after 10% weight loss [53]. The instructions were to eat until comfortably satisfied on two non-consecutive days and to continue eating until stopped by remotely administered verbal instructions to make ratings after every 150 g (7 times). A formula diet, mixed in the laboratory, was the test meal, and its palatability was less than optimal (mean rating = 45/150 mm = 30%). There was no effect of the treatments on intake, which was higher during the fixed meal (1040 kcal ± 41 SE) than during the meal eaten to feel comfortably satisfied (849 kcal ± 41 SE). However, at the ends of each meal participants reported they felt they had eaten significantly more and felt fuller after, than before, leptin.

**Fig. 1:**
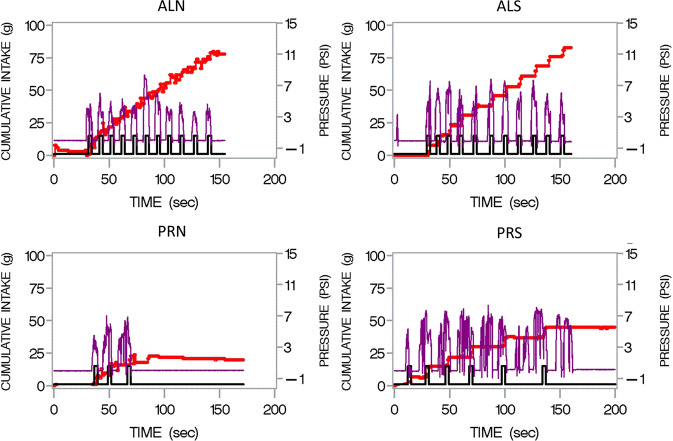
“Sipograph” Graphical display of intake (red line) reinforcement (black line) and pressure exerted (purple line) by participant consuming from the sipometer [55] under continuous reinforcement (AL) for 2 min (upper panels) or progressive ratio (“PR”) for unlimited time (lower panels) when the reinforcer was either a non-sweetened (N left) or sweetened (S right) Kool Aid. The pressures are greatest when the participant was sipping on the progressive ratio schedule for the sweet as compared to the non-sweet beverage. The challenge here is to quantify these pressure waves so that individuals and beverages can be compared.

*Sipometer and microstructure (2007–2020)*: The latest application of the UEM has been to couple it with an automated sipping device, that we call the “sipometer”. In order to translate animal models of motivation and reward to humans, Anthony Sclafani, who had been using a progressive ratio licking reinforcement paradigm [54] to measure responses to sweet and fatty liquid foods, constructed a sipometer, which I tested in humans starting in 2007. Three studies were conducted with it [55]; it has potential application for tests of motivational impairment (e.g., excessive motivation) in obesity and for individualized approach to diagnosis and treatment of eating disorders. Deprivation and increased liking and wanting were reflected in greater pressure applied to obtain the reinforcer. Another advantage of the sipometer is that it can be used to generate clusters and bursts that have recently been proposed as human analogues to licking patterns in rats [56]. Figure 1 illustrates the challenge that the instrument poses for simultaneous assessment of motivation and microstructure of consummatory behavior. This challenge is analogous to the analytical challenges posed by early measurements of chewing and swallowing from the edogram [57].

## Additional related work of other laboratories

The UEM has been replicated with novel applications in many other laboratories to address a diverse collection of research questions. Table 1 provides a brief summary of these questions which fall into five categories: (1) Use of the device to retrain eating behavior (lines 1,2); (2) Confirmation and extension of earlier findings on relation of eating rate to other variables, such as palatability, energy density, eating disorder (lines 3,4); (3) consistency and different methods of analysis of results (lines 5,6); (4) effects of a variety of manipulations on eating rate and amount consumed (lines 7,8); and (5) awareness of monitoring (line 9). All of these results contribute to the sense that the UEM is a widely used and validated technique for measuring intake episodes which are the building blocks needed for understanding the mechanisms of energy intake control and its influence on body weight.

**Table 1:** UEM Research.

Laboratory/reference	Research question	Outcome
1. Ford, Shield, Sodersten [66]	Does modifying eating behavior with a feedback device (mandometer) facilitate weight loss in adolescents with obesity?	Monitoring significantly lowered mean BMI SDS at 12 months compared with standard care.
2. Södersten [65]	Does provision of feedback to control rate of eating assist with intake and weight control?	Provision of visual feedback on the computer screen that the subject can adapt to control eating rate enabled restoration of weight and health in patients with both anorexia and overweight.
3. Westerterp [8, 76]	Which is more important in determination of cumulative intake curve parameters, energy density, or volume/weight?	In the short run deceleration is higher the smaller the energy density, but no different when deceleration is expressed as energy [76].
4. Yeomans [10]	What is regulated, volume or energy?	People tend to regulate the mass (or volume) they consume rather than energy intake.
5. Martin [77]	How consistent are results over time?	Measures of food intake were stable for men and women, regardless of sandwich variety.
6. Dovey [20]	What is the effect of different methods of analysis on stress response to fullness from cumulative intake curve?	the coefficient approach found a significant difference in the fullness curves between relaxation and cold pressor conditions (*p* = 0.012), due to the presence of a quadratic component in the cumulative intake curve in the stress condition which was not present in control (*p* = 0.017).
7. Barkeling [78]	Is protein more satiating than carbohydrate?	Following high protein and high carbohydrate lunches, subjects ate less only during the first quarter of an evening meal, after the high protein than after the high carbohydrate lunch.
8. Rossner–Blundell [37]	What is the effect of GLP-1 infusion on cumulative intake curve and intake?	Intake was reduced by 21% after GLP-1 compared to saline infusion but neither initial rate nor deceleration was affected. Overall eating rate was lower after GLP-1.
9. Thomas et al. [79]	Does awareness of eating being monitored affect consumption.	Awareness of the UEM affected cookie, but not pasta consumption.

*Validation of other measures with the* UEM: The UEM has also been instrumental in the validation of newer techniques for measurement of eating behavior. Mattfield [58] described an application in which a bit counter [59] worn on the wrist was validated as a measure of eating rate and bite size, by placing the bite counter on participants who were also eating from a UEM. The device detected 90% of the bites detected by the UEM. In another attempt to compare eating rates under field and laboratory conditions participants in an eating behavior study estimated their rate of eating under both conditions. Actual eating rates were measured via food diaries and in the laboratory with a UEM. Self-reported eating rates cohered with UEM reported eating rates when the rates were stratified into three groups. However, only rates of lunches and not dinners, snacks, or breakfasts appeared to cohere with self-reported eating rate (SRER). Differences in accuracy of recording eating rate between food diaries with different items and a UEM with a uniform food could have prevented coherence between rate measures, but the agreement of SRER with UEM eating rate indicated that the SRER was a valid measure of eating rate.

*Other clinical applications and pharmacology*: The technology of the UEM has been coupled with training of eating rate [60] in adolescents with eating disorders along with cognitive behavioral therapy that combines specific instructions with feedback provided on eating rate. The treatment program has been a great success [61–63]. The identification of patients whose eating rates do not decelerate has also been useful in developing appropriate therapeutic approaches. Linear eaters have difficulty maintaining their intake when eating rate is dissociated from its baseline level and this puts them at risk of developing disordered eating. Feedback on eating rate can therefore be used as an intervention to treat eating disorders [64, 65]. Fitting of a sigmoid curve to the satiation data [64] has been helpful in both treatment of eating disorders and obesity [62] and for understanding the relationship of satiation development to rate of eating [64] and treatment of childhood eating problems [66].

The UEM has also been instrumental in testing the effects of drugs particularly on changes in appetitive ratings per unit eaten during the course of a meal [67–69]. Finally, alcohol has been shown to provide an aperitif effect. It does not necessarily change initial eating rate, but increases hunger ratings and raises overall rate of eating [70, 71].

## Strengths, limitations, and future considerations

The major strength of the UEM in comparison to questionnaires, food diaries, remote observation, photography of eating, or wearable software is that the actual rate of intake (convertible to energy units) in physical standardized units, not an estimate, can be made as individuals consume either solid or liquefied food. That this rate is ultimately under physiological control has been demonstrated as described above in relation to nutrient density, physiological variables, such as gastric distention and hormone administration, and hedonic ratings, exercise, and social activity. The rate of eating can be treated as a physiologically controlled variable, analogous to other such variables like heart rate, respiratory rate, clearance of metabolites, gastric emptying rate, rate of nutrient absorption, and as such can be used as an indicator of health, disease, or disorder. It has already been shown to predict metabolic disturbances in severe obesity [72], and thus has the potential to reveal other physiological disturbances.

Other measures related to the rate of eating are indirect. Measurement of chewing or swallowing activity or number of bites, cannot be directly translated into nutrient or volume intake. Ultimately, the mechanisms that underlie body weight control and whether or not obesity occurs, must rely on the rates of energy expenditure and intake (i.e., the rate of energy intake, which is the integral of the chewing and swallowing amounts converted to energy over time), not how many chews or swallows occur. On the other hand, these methods are valuable in their own right because the neural controls that facilitate or inhibit nutrient consumption must operate on a neuro-muscular pathway which is better studied by direct measures of its output. Consequently, a correlation of micro-behavior units with rates of energy consumption will be needed for a complete model of the control of intake and body weight. An alternative mechanism also exists in that peptides that control food intake are also affected by the rate of eating [73].

Cognitive controls of intake, such as beliefs, attitudes, and habits also contribute to food intake control, and the potential influence of these variables raises concern about the interpretation of intake data. Amounts eaten in response to instructions to either eat until comfortably satisfied, or what you would usually eat in this situation assume that there is a controller for these states that is recognized by the individual, and is analogous to what happens when an animal displays behaviors that indicate it has had enough (the satiety sequence). Booth [74] has argued that termination of a meal is a judgment process similar to filling out a scale. Because termination of a meal is subject to judgment rules, instructions about test meals and cognitive responses to them must include comparison to standards and the ability to discriminate among intensities of stimuli. When intakes without comparison to standards vary from trial to trial in an uncontrolled way, they are more variable than if the instructions related intake to a previously experienced test the subject was told to use as a standard. It is possible that subjects in fact behave as though an adaptation meal in the laboratory was a standard even without such instructions. In the future, investigators should try to incorporate standardized contexts or eating situations as referents to the particular manipulation whose effects they wish to measure.

The major limitation of the UEM and test meal paradigm is that it measures intake of a single item (or in some instances multiple items) at a single time point under a single condition, and that whatever leaves the scale goes into the subject’s mouth with only minor delay. Potential confounds could occur if heating or cooling of the item changed its weight. In order to generalize findings, repeated measures are necessary, and we have shown that reproducibility is within 15% across repeated trials at the same time of day [6]. However, whether results of experimental treatments persist over time and at different times and under different conditions will require additional research, and applications that move the test meal from the laboratory to field, but retain its precision. Single tests of eating behavior also suffer from the fact that eating in a laboratory situation is unusual, and any mechanistic test must include adaptation of at least one day, and depending on the severity of an invasive manipulation, such as IV infusion of test product, two or more days [75]. For tests where adaptation is impractical, results must be interpreted with caution.

For future consideration, a program of standardized testing across laboratories to measure rate of eating in the same physical (as opposed to observational) units should be developed. Such a program would enable valid and non-confounded comparisons across cultures, foods, and potential pathologies, with the establishment of norms. At the present time, both the scales for measurement of sensations and perceptions, and the instruments for measurement of actual physical consumption are confounded by differences in items eaten, instructions to participants, time of day, and other variables. If a consortium of laboratories received funding for development of common methods, these problems could be solved.

## Conclusions

The UEM and laboratory test meal are valid measures and standardized procedures that assess energy intake at a point in time, just as physiological tests for energy expenditure, and a host of other physiological functions are used in their respective domains. Like those tests, they are subject to the limitations of the laboratory. They are, nevertheless, vital tests. Just as conditioned reflexes were not discovered on a busy street corner, *mechanisms* that control energy intake will only be discovered when intakes are subjected to laboratory precision. Unless one puts the individual on a scale and does continuous weighing while the individual eats, there is no other way to get a valid, accurate, and precise measure of the microstructure of amount consumed as a rate over time. Wearable software, movement, muscular detection, and cameras notwithstanding, only weighing the disappearance of the food or beverage as it is consumed will provide such a measure.
